# Giant Left Atrial Thrombus: A Source of Systemic Emboli

**DOI:** 10.1016/j.case.2021.12.005

**Published:** 2022-01-25

**Authors:** Ziad Arow, David Pereg, Abid Assali, Yoram Neuman

**Affiliations:** Department of Cardiology, Meir Medical Center, Kfar Saba, Israel

**Keywords:** Case report, Systemic embolization, Anticoagulation, Thrombosis, Echocardiography

## Abstract

• It is important to maintain therapeutic-range INR after mechanical valve replacement.
• A normally functioning mechanical mitral valve does not preclude a large LA thrombus.
• There is a high index of suspicion of LA thrombus in patients with systemic embolization.

## Graphical abstract

**Figure:**
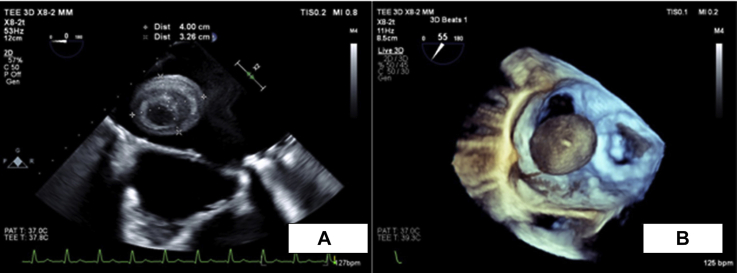

## Introduction

Mechanical heart valves require lifelong treatment with vitamin K antagonist and close international normalized ratio (INR) follow-up. Target INR is determined mainly by prosthesis thrombogenicity and patient-related risk factors.^1^ Subtherapeutic anticoagulation is a major risk factor for mechanical valve thrombosis and is associated with serious adverse clinical outcomes.^1^^,^^2^ Therefore, patient education is very important for achieving therapeutic-range INR.

We present a case of systemic embolization due to a free giant left atrial (LA) thrombus in a middle-aged man with a mechanical mitral valve and poor compliance to vitamin K antagonist.

## Case Presentation

**Figure 1:**
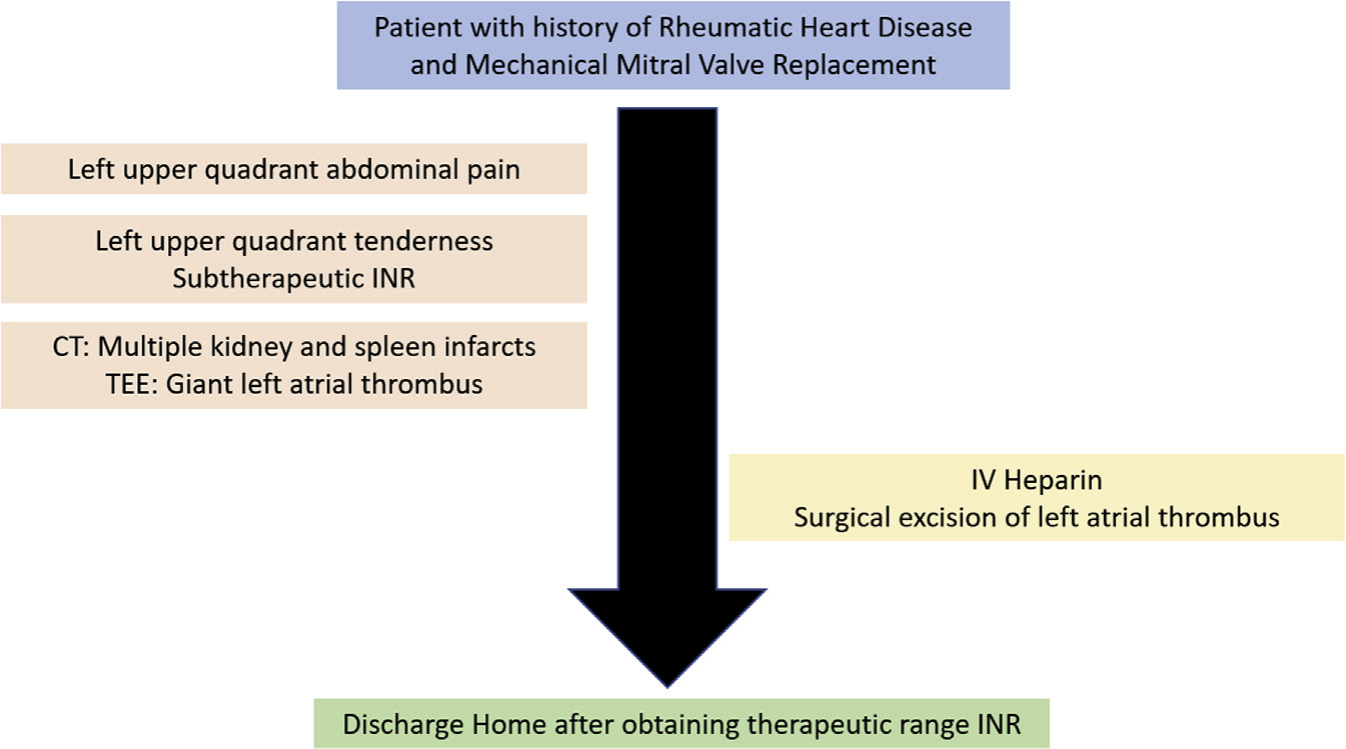
Case timeline of patient.

**Figure 2:**
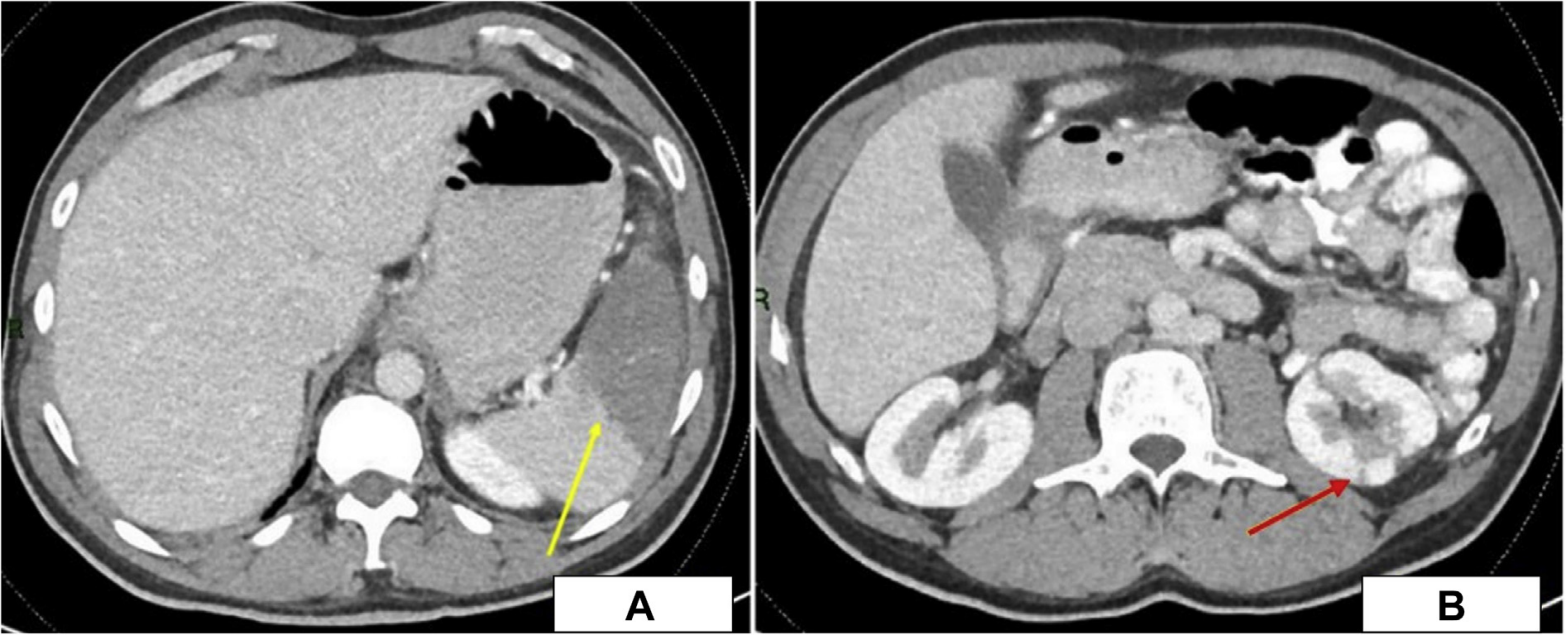
Abdominal computed tomography axial view showing **(A)** splenic infarct (*yellow arrow*) and **(B)** left renal infarct (*red arrow*).

**Figure 3:**
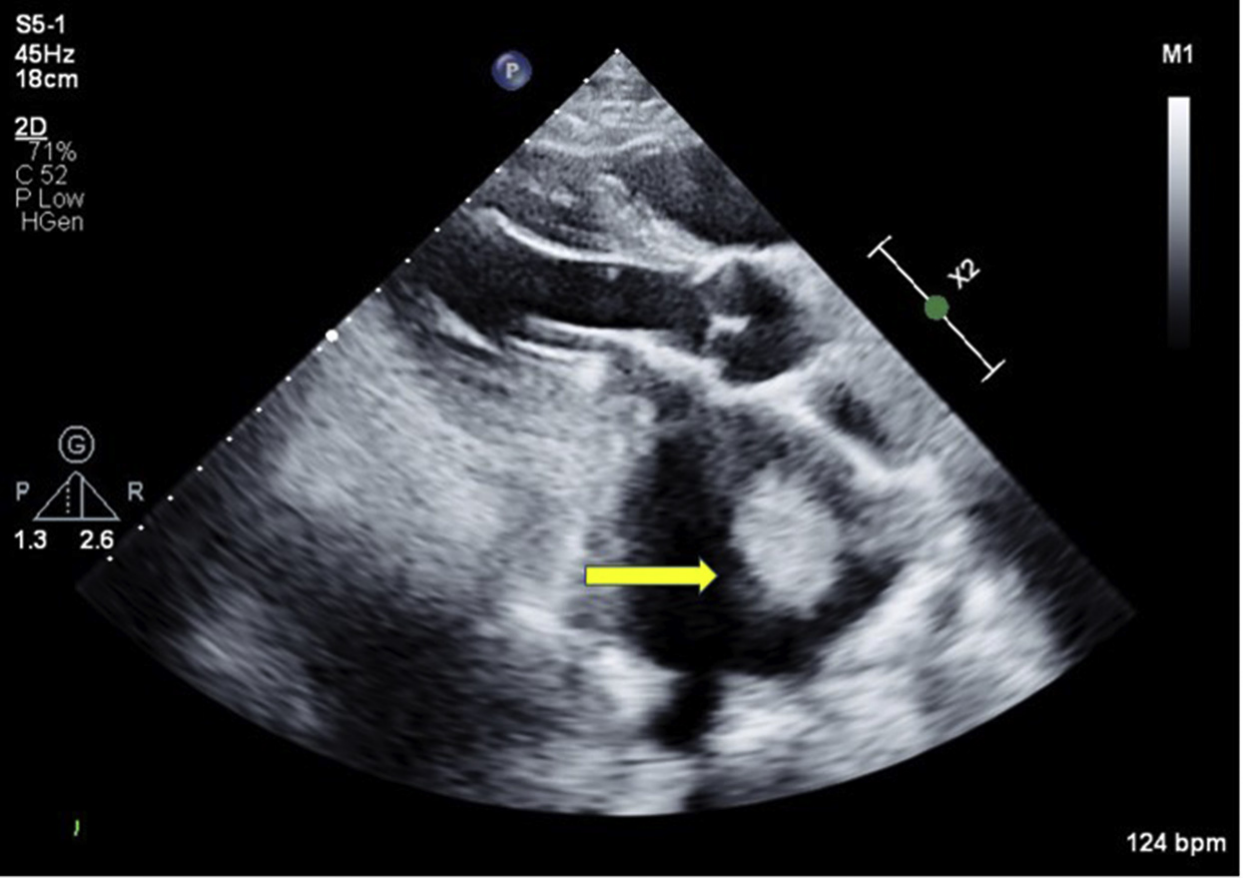
Transthoracic echocardiography: two-dimensional parasternal long-axis view showing LA mass (*yellow arrow*).

**Figure 4:**
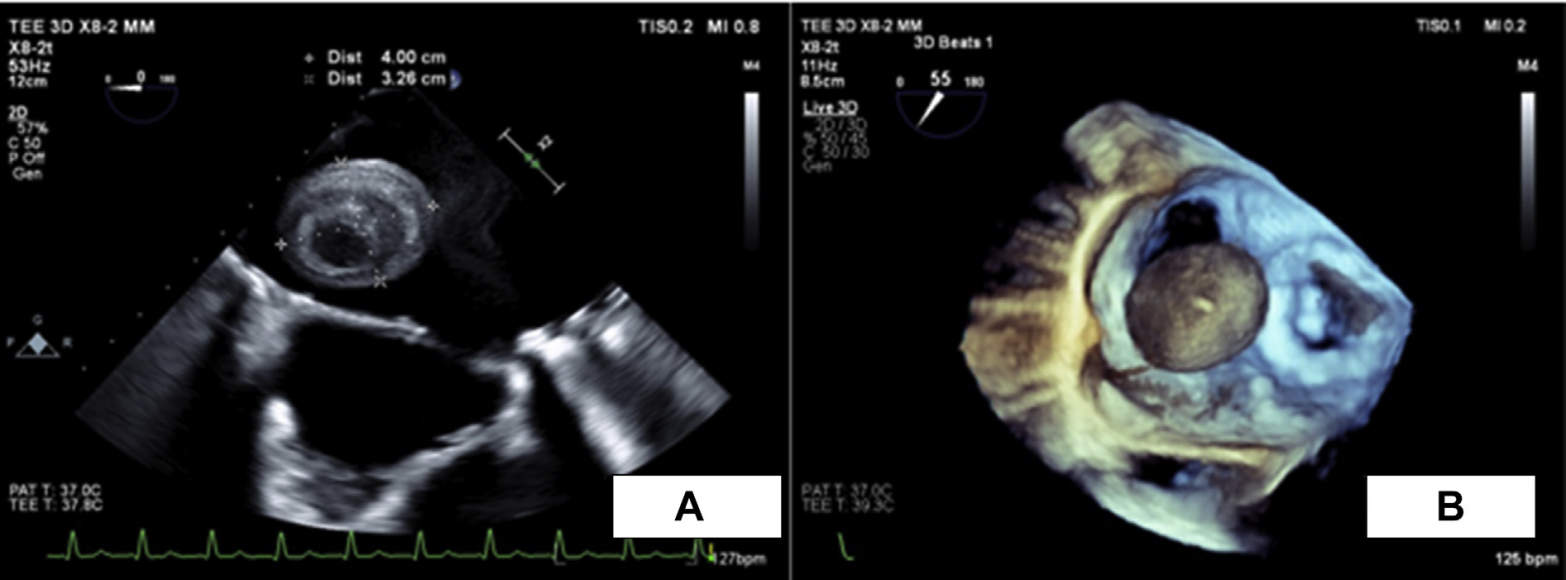
**(A)** Transesophageal echocardiography: two-dimensional midesophageal view showing giant LA thrombus. **(B)** Transesophageal echocardiography: three-dimensional zoomed midesophageal view demonstrating LA thrombus.

**Figure 5:**
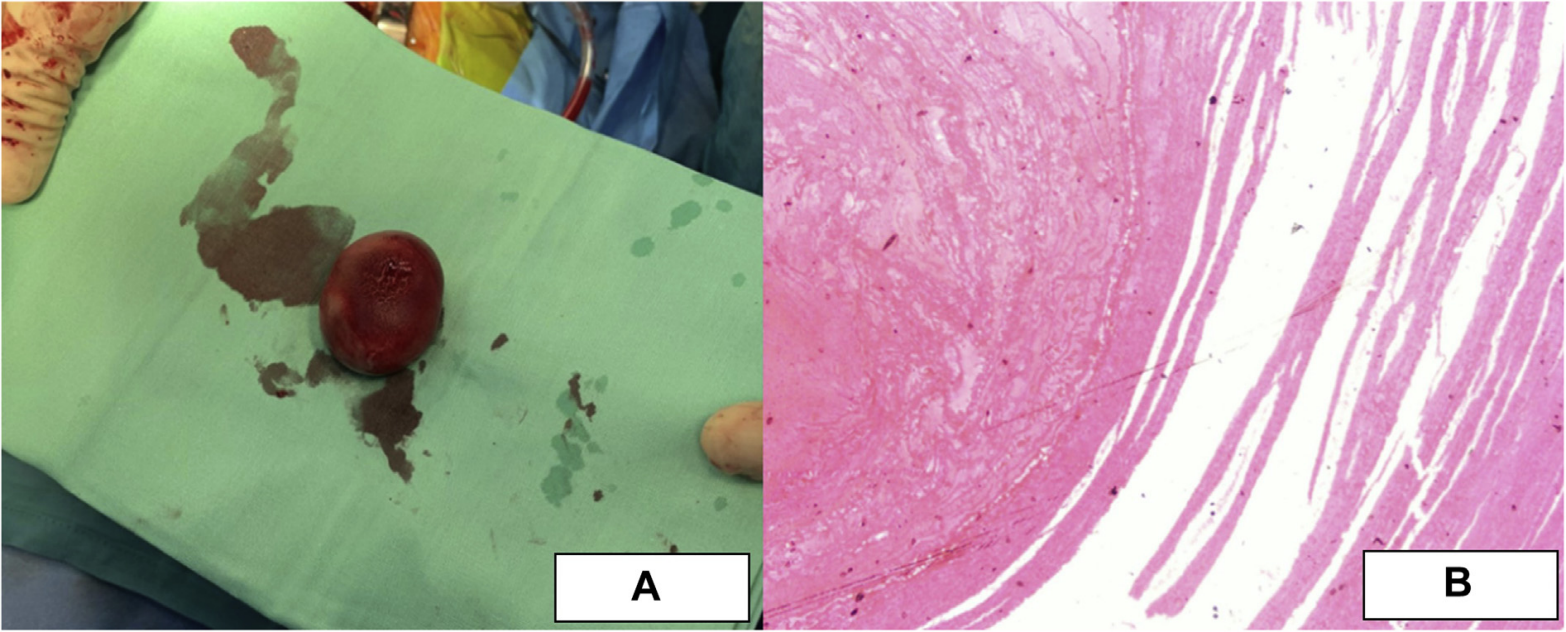
**(A)** Giant LA mass macroscopic appearance after surgical removal. **(B)** Pathological evaluation showing concentric deposition of thrombus composed of nuclear debris, fibrin, and erythrocytes.

A 49-year-old man presented to the emergency department with left upper quadrant abdominal pain in the past 3 days. The patient had recently stopped all his chronic medications, including warfarin within the past 4 months. In his past medical history, the patient underwent mechanical mitral valve replacement (MVR; Sorin Biomedica 31 mm, Saluggia, Italy) 20 years ago due to severe rheumatic mitral stenosis (Figure 1). His medical history also includes atrial fibrillation, hyperlipidemia, and hypertension. Chronic medical therapy included warfarin, ramipril, simvastatin, and sotalol. Upon admission, his vitals included irregular tachycardia of around 110 beats/minute and normal oxygen saturation. Cardiac auscultation demonstrated irregular normal mechanical valve sounds. His lungs were clear without any signs of heart failure. Abdominal palpation demonstrated left upper quadrant tenderness without peritoneal signs. Laboratory findings were remarkable for mild anemia (Hgb, 10 g/dL) and subtherapeutic INR of 1.3. His white blood cell count, creatinine, arterial blood gases, and lactate were normal as was his urinalysis. An electrocardiogram showed atrial fibrillation with no signs of acute myocardial ischemia. Abdominal contrast computed tomography showed splenic infarct and multiple small renal infarcts (Figure 2). Transthoracic echocardiography (TTE) showed moderate left ventricular systolic dysfunction (ejection fraction, 38% by Simpson's method) with normally functioning mechanical mitral valve, normal transmitral gradients (average mitral valve mean gradient, 4 mm Hg), and severe left atrium enlargement (left atrium diameter, 6 cm) with suspected LA mass (Figure 3, Video 1). Fluoroscopy was performed next and ruled out a stuck mechanical mitral valve (Video 2). For better visualization, evaluation, and characterization of LA mass seen on TTE, transesophageal echocardiography (TEE) was performed and revealed a free, mobile, hyperechoic, 3 × 4 cm thrombus in the left atrium (Figure 4, Videos 3-5). Treatment with intravenous heparin was initiated immediately. Following a heart team and a patient-informed discussion, given the extreme thrombus size, the evidence of recurrent embolization, and the risk of mechanical valve obstruction under anticoagulation-only therapy, the recommended treatment was surgical excision of the LA mass. The patient gave his informed consent and was transferred to cardiac surgery, where he underwent successful removal of the giant free LA mass (Figure 5A). Pathological evaluation confirmed the clinical diagnosis of LA thrombus (Figure 5B). The postoperative course was uneventful, and the patient was discharged home on treatment with warfarin. Discharge home followed several detailed explanations to the patient and his family regarding the importance of anticoagulation and maintaining an INR of 3.^1^

## Discussion

Patients with mitral valve stenosis secondary to rheumatic heart disease including those following mechanical MVR are at increased risk of thrombotic and cardioembolic complications.^3^^,^^4^ The thrombotic risk is further increased in the presence of atrial fibrillation, which coexists in the majority of cases. Patient education regarding the critical importance of warfarin adherence and maintenance of adequate anticoagulation, as well as close follow-up and monitoring, is extremely important following mechanical heart valve replacement. In such patients, and especially when subtherapeutic anticoagulation is suspected, a clinical presentation with systemic embolization should mandate an urgent assessment for mechanical valve and LA thrombosis. In most cases, this assessment must include TEE since TTE may not be sensitive enough.^4^, ^5^, ^6^ Furthermore, we find it important to highlight that a normally functioning mechanical mitral valve does not preclude the presence of LA thrombus.^7^^,^^8^

## Conclusion

In patients with a mechanical MVR presenting with systemic embolization, a high index of suspicion for a cardiac source of emboli and strategic incorporation of multimodal cardiac imaging is warranted. This often requires TEE to optimally visualize the left atrium, which may be shielded from the MVR with TTE alone. Early diagnosis and treatment of LA or mechanical valve thrombosis can be lifesaving and prevent further cardioembolic events.

## Supplementary Data

Video 1 Transthoracic echocardiography: two-dimensional parasternal long-axis view showing severely dilated LA cavity with a suspected LA mass.

Video 2 Fluoroscopic imaging of the mechanical mitral valve taken at the 43 right anterior oblique/35 caudal view demonstrating normal motion of both disks.

Video 3 Transesophageal echocardiography: two-dimensional midesophageal view showing thrombus floating in the left atrium and colliding with the mitral valve.

Video 4 Transesophageal echocardiography: two-dimensional midesophageal view showing hypercoagulable state with thrombus formation in the left atrium.

Video 5 Transesophageal echocardiography: three-dimensional midesophageal view showing giant LA thrombus.
